# Supersize me: A seed-specific protein facilitates lipid droplet enlargement in Arabidopsis

**DOI:** 10.1093/plcell/koaf131

**Published:** 2025-05-21

**Authors:** Vicky Howe

**Affiliations:** Assistant Features Editor, The Plant Cell, American Society of Plant Biologists

In plants, lipid droplets (LDs) are an important energy store for seedlings in the early post-germinative growth stage, before the plant can photosynthesize. LDs are formed when hydrophobic triacylglycerols are packed into a phospholipid monolayer that buds from the ER. Embedded in the phospholipid membrane is a complex array of proteins that orchestrates LD assembly and regulates their cell-specific biophysical properties and functions. OLEOSINs (OLEOs) are the most abundant LD proteins in seeds and are co-translationally synthesized at the ER to be incorporated into nascent LDs (Abell et al. 2002). OLEOs play a role in LD stabilization by preventing LD-LD fusion and protecting triacylglycerols from lipases, particularly in response to seed desiccation and freezing winter temperatures (Shimada et al. 2008). However, OLEOs are rapidly degraded upon seed germination, destabilizing LDs and allowing the stored fats to be broken down and utilized by the growing seedling (reviewed in Zienkiewicz and Zienkiewicz 2020) (Figure).

**Figure.**
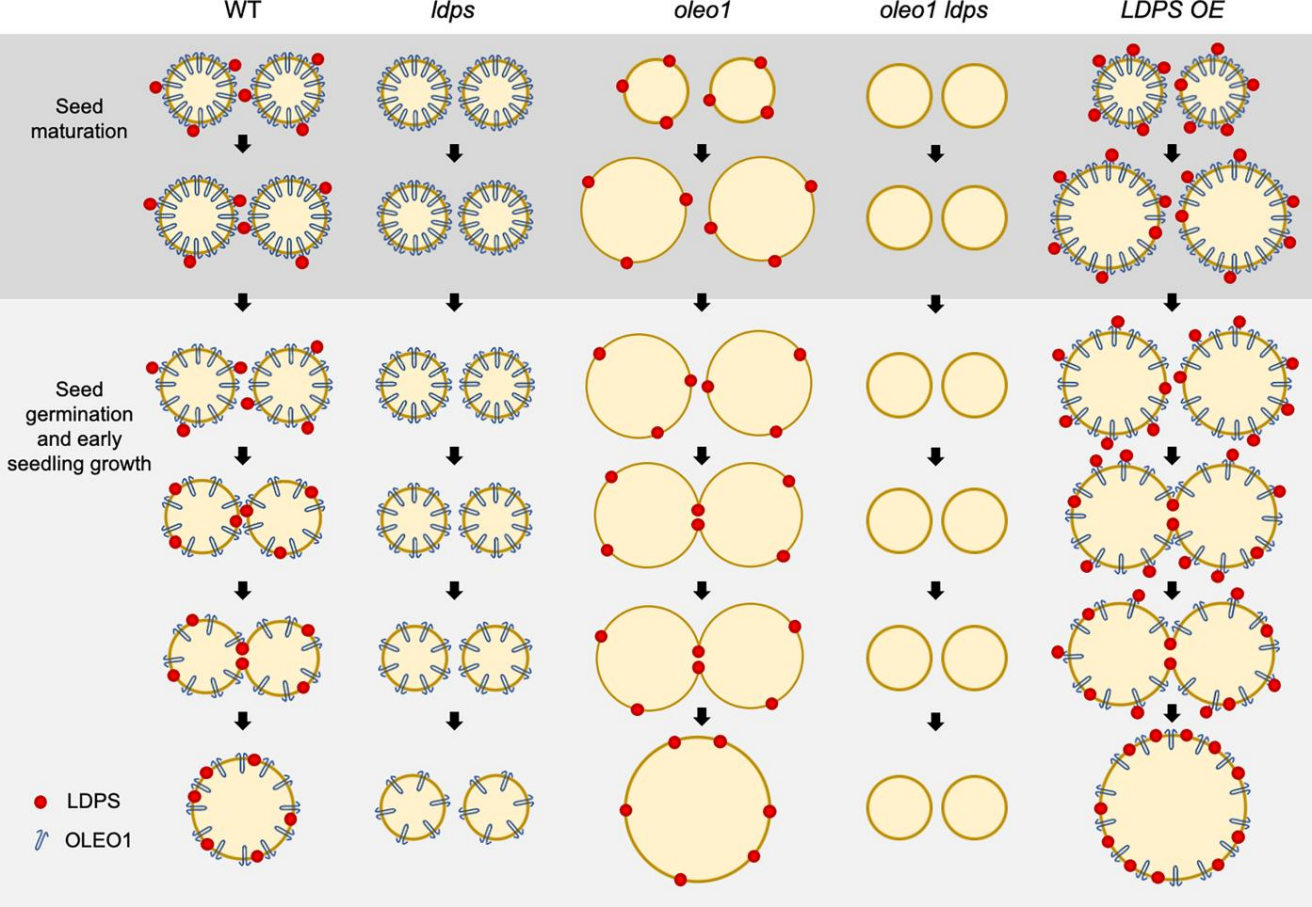
LPDS and OLEO1 share roles in modulating LD size. Under normal conditions in the wild type (WT), LDPS is low in abundance, tethered to the LD by interaction with high-abundance OLEO1, which acts to prevent LD-LD fusion in maturing seeds. Post germination, OLEO1 is degraded and LDPS associates with the LD membrane, promoting LD-LD fusion and increasing LD size. In *ldps* and *oleo1 ldps* mutants, LDPS is not there to promote LD-LD fusion and lipid droplets remain small. In the absence of OLEO1 (*oleo1*) or when LDPS expression is increased relative to OLEO1 (LDPS OE), LDPS (together with other unknown seed-specific factors) promotes LD-LD fusion, resulting in supersized lipid droplets. Figure reprinted from Doner et al. 2025, Figure 12.

While OLEO content may influence the size of nascent LDs (Siloto et al. 2006), the mechanisms controlling LD size and function are not well understood in plants, and recent studies have identified even more plant-specific LD proteins whose roles have yet to be characterized (Kretzschmar et al. 2020). One such protein, the focus of recent work by Nathan M. Doner and coauthors (Doner et al. 2025), is LIPID DROPLET PROTEIN OF SEEDS (LDPS). In Arabidopsis, LDPS is expressed exclusively in mature seeds and young seedlings in the first few days after germination, and Doner and colleagues identified the region of LDPS that allows it to specifically localize to LDs, likely via protein-lipid interactions.

To investigate what role LDPS plays in regulating LDs, the researchers compared lipid droplets in seeds and young seedlings of wild-type and *ldps* Arabidopsis mutants. Although there was no difference in LD size in *ldps* mutant seeds, in seedlings 2 days post-germination, LDs were much smaller compared to the wildtype and remained small throughout the early post-germinative growth stage. Conversely, overexpressing LDPS resulted in the formation of “supersized” LDs in post-germinative seedlings (Figure). This finding suggests that LDPS has a function in controlling LD size, perhaps by promoting LD-LD fusion.

In search of a mechanism by which LDPS might regulate LD size, the group next looked for LDPS interaction partners. Using a mating-based yeast split-ubiquitin system, they identified OLEO1. Indeed, they showed that in *N. benthamiana* leaves, OLEO1 was able to recruit a mutant LDPS (lacking a LD targeting signal) to LDs, tethering it via protein-protein interactions. Given the apparently opposing functions of OLEO1 and LDPS in regulating LD size, the group generated a range of Arabidopsis lines with various combinations of LDPS and OLEO1 knockout and overexpression to investigate how different ratios of the 2 proteins might affect LD size. They further subjected seeds to freezing treatment to induce biophysical stress that might trigger LD-LD fusion. Just like in previous studies (Shimada et al. 2008), loss of OLEO1 resulted in larger LDs in early post-embryogenic seedlings, and this was exaggerated by freezing treatment. Overexpressing LDPS in a wild-type background, where OLEO was still present, also resulted in larger LDs that modestly increased in size upon freezing. However, the biggest difference was seen in lines overexpressing LDPS in an *oleo* background, where LDs were similar in size to the freeze-treated *oleo* mutants and freezing treatment further increased LD size.

This suggests that LDPS and OLEO1 have opposing roles in modulating LD size that are dependent on the relative ratios of the 2 proteins. The authors propose that OLEO1 recruits LDPS during LD biosynthesis, with LPDS held to the LDs by protein-protein interactions in the crowded protein environment of the lipid membrane. OLEO1 stabilizes LDs and prevents TAG lipolysis, conserving the seed's energy stores. However, as OLEO1 is degraded post-germination, LDPS gains access to the membrane and can associate with it via its LD-targeting signal. Then, in OLEO's absence, LDPS promotes LD-LD fusion. The destabilized LDs can subsequently be accessed by lipases, providing energy to the growing seedling (Figure).

Interestingly, overexpressing LDPS in Arabidopsis in a wild-type background slightly increased the overall lipid content of the seeds. While seeds lacking OLEOs have larger LDs, they are less resilient in terms of desiccation and freezing tolerance (Shimada et al. 2008). However, Arabidopsis lines overexpressing or lacking LDPS did not appear to be disadvantaged under normal growth conditions. This raises the question as to whether manipulating levels of proteins involved in increasing LD size could increase yields in oilseed crops and warrants further investigation.

## Recent related articles in *The Plant Cell*

- Zhuang et al. 2024 reviewed the key innovations and studies over the past century that enabled the visualization, identification, and characterization of the elusive organelles of the plant endomembrane system.
- Nguyen and Nakamura 2023 identified a pair of differentially localized lipid phosphate phosphatases that mediates membrane lipid biosynthesis in Arabidopsis pollen and seeds and requires inter-organelle communication.
- Luzarowska et al. 2023 studied lipid remodeling under carbon starvation, identifying a branch point in fatty acid biosynthesis.

## Dive Curated Terms

The following phenotypic, genotypic, and functional terms are of significance to the work described in this paper:

LDPS Gramene: AT3G19920
LDPS Araport: AT3G19920
